# Analysis of disparities in medical check-ups for patients with diabetes and hypertension: associated factors and trends in a nine-year national survey

**DOI:** 10.3389/fcdhc.2025.1714205

**Published:** 2026-01-09

**Authors:** Víctor Juan Vera-Ponce, Fiorella E. Zuzunaga-Montoya, Félix García-Ahumada, Darwin A. León-Figueroa, Percy Díaz Morón, Mario J. Valladares-Garrido

**Affiliations:** 1 Facultad de Medicina (FAMED), Universidad Nacional Toribio Rodríguez de Mendoza de Amazonas (UNTRM), Chachapoyas, Peru; 2 Universidad Continental, Lima, Peru; 3 Escuela de Medicina Humana, Universidad Señor de Sipán, Chiclayo, Peru; 4 Facultad de Medicina Humana, Universidad de San Martín de Porres, Chiclayo, Peru; 5 EpiHealth Research Center for Epidemiology and Public Health, Lima, Peru

**Keywords:** diabetes, hypertension, disparities, tertiary prevention, vision screening, arterial pressure, glycemic control

## Abstract

**Introduction:**

Diabetes and hypertension (HTN) are non-communicable chronic diseases that pose a significant challenge to global public health. However, substantial disparities in the performance of these essential check-ups among diagnosed patients have been identified.

**Objective:**

1) To determine the existing disparities in the check-ups of patients with diabetes and HTN, 2) To observe the trend of these check-ups over the years through a Peruvian national survey.

**Methods:**

A cross-sectional analytical study using information from Peru’s Demographic and Family Health Survey between 2014 and 2022. The main variables were performance in the last year of ophthalmic, blood pressure, and glucose check-ups.

**Results:**

Regarding the prevalence of check-ups in the last year, it was high for ophthalmic examinations (HTN: 65.46%, diabetes: 70.54%), blood pressure measurements (HTN: 81.82%, diabetes: 79.92%), and glucose measurements (HTN: 56.72%, diabetes: 83.76%). In the trend analysis for patients with diabetes, minimal variation was observed between the evaluations from 2014 to 2019, with a notable decrease in 2020 and 2021, particularly in ophthalmic check-ups, followed by a recovery in 2022. The most consistent determinants of check-up performance across both conditions were older age (≥60 years), higher educational level, higher socioeconomic status, and having health insurance. Female sex was associated with higher check-up rates in patients with HTN. Geographic and ethnic disparities were also observed, with urban residents and certain ethnic groups showing different check-up patterns.

**Conclusions:**

This study has revealed significant disparities in the performance of essential health check-ups among Peruvian patients with diabetes and HTN, showing that various determinants play a crucial role in the frequency of these check-ups.

## 1 Introduction

These two widespread non-communicable chronic illnesses, diabetes and arterial hypertension (HTN) present a tremendous global public health obstacle. The World Health Organization (WHO) projects that by 2040, over 642 million individuals worldwide will have diabetes, a stark increase from the current figure of more than 422 million people affected by the disease. Likewise, it is estimated that HTN currently affects over a billion people worldwide, with forecasts anticipating that number will rise to approximately one and a half billion by the middle of the next decade. The rapidly increasing statistics regarding chronic conditions pose worries for both industrialized and emerging nations alike as the weight of dealing with such long-term illnesses perpetually expands (1, 2). The rising tide of diabetes and HTN cases highlights the pressing importance of developing competent management tactics and initiatives intended to alleviate the strain imposed on individuals and medical organizations by these widespread conditions.

Once diagnosed with diabetes or hypertension, patients must undergo regular clinical check-ups to monitor key health indicators such as blood pressure, fasting glucose, and visual health (3, 4). While routine check-ups prove pivotal to the astute handling of long-term afflictions, their significance is further emphasized in forestalling or postponing the emergence of hazardous consequences. Careful tracking of both blood pressure and glucose levels through consistent checkups is paramount for medical professionals to properly oversee patients’ regimens and tweak treatments as needed for those with diabetes to avoid dangerous blood sugar fluctuations or episodes of erratic glycemic symptoms in those managing hypertension. Furthermore, visual check-ups are critical given the high risk of diabetic or hypertensive retinopathy (5, 6). These monitoring practices, recommended by international guidelines, underscore the importance of proactive health management by patients and healthcare providers.

However, significant disparities have been identified in the performance of these essential clinical checks among patients diagnosed with diabetes and hypertension. Despite clear recommendations for regular monitoring, recent studies have revealed that many patients go more than a year without undergoing the necessary blood pressure, fasting glucose, and visual exams (7–10). These gaps in medical management can lead to inadequate management of these chronic diseases and increase the risk of severe complications. In Peru and Latin America, evidence regarding compliance with recommended medical check-ups among patients with diabetes and hypertension remains limited, with few population-based studies systematically evaluating temporal trends and associated sociodemographic factors that influence healthcare utilization in these populations. Recognizing these disparities highlights the critical need to investigate and address the underlying factors contributing to the lack of regular medical follow-up. In this context, the aim of our study is 1) to determine the existing disparities in the medical management of patients with diabetes and hypertension and 2) to observe the trend of these check-ups over the years through a Peruvian national survey. This approach will allow us to identify critical areas for intervention and improve health outcomes for this vulnerable population.

## 2 Methods

### 2.1 Study design and context

This study used data from the Peru Demographic and Family Health Survey (ENDES in Spanish), a nationally representative household survey conducted annually by the National Institute of Statistics and Informatics (INEI). ENDES employs a probabilistic, stratified, two-stage sampling design that differentiates between urban and rural areas across Peru’s 24 departments, ensuring representative coverage of the Peruvian population (11). The survey collects information on health status, healthcare utilization, and sociodemographic characteristics through face-to-face interviews conducted by trained personnel. Anthropometric measurements, including height and weight, are obtained directly by field staff using standardized protocols. Further details on ENDES methodology are available in the technical documentation provided by INEI. The STROBE guidelines were followed to ensure the study’s quality and transparency (12).

### 2.2 Population, eligibility criteria, and sample

The original study focused on Peruvian individuals aged 15 to 99 years, considering both urban and rural areas of the country’s 24 departments. ENDES’s sampling methodology was based on a probabilistic, stratified, two-stage design differentiating urban and rural areas.

For the present study, only participants who indicated having been diagnosed with diabetes and HTN were selected. We restricted the analysis to individuals aged 50 years and older because ENDES systematically collects information on ophthalmic examinations only from this age group. Patients could be included in both the diabetes and hypertension groups if they reported having both conditions; thus, these groups are not mutually exclusive.

### 2.3 Variables and measurement

The main variables were three:

#### 2.3.1 Ophthalmic check-up

Assessed by asking, “How long ago was the last time you had your vision checked or measured?” The response was dichotomized into less than one year and one year or more. This variable measures access to and utilization of eye care services, which is the primary focus of our study when examining disparities.

#### 2.3.2 Blood pressure check-up

Assessed by asking, “In the last 12 months, has a doctor or other health personnel measured your blood pressure?” The response was dichotomized into yes versus no.

#### 2.3.3 Glucose check-up

Assessed by asking, “In the last 12 months, has a doctor or other health personnel measured your glucose or blood sugar?” The response was dichotomized into yes versus no.

These monitoring timeframes align with international clinical guidelines, as recommended by the American Diabetes Association. The Association recommends annual dilated eye examinations for all adults with diabetes (3), blood pressure assessment at every routine diabetes visit (3), and glucose monitoring at least twice yearly for those meeting treatment goals (3). The European Society of Cardiology/European Society of Hypertension guidelines recommend blood pressure measurement at every clinical visit and annual fasting glucose screening in patients with hypertension to assess cardiovascular risk (6, 13, 14).

Several sociodemographic and clinical characteristics were examined as independent variables. Sex was classified as female or male. Age was categorized into two groups: 50–59 years and 60 years or older (Age group). The region of residence was classified according to ENDES geographic divisions as Metropolitan Lima, the Rest of coast, the Mountain Range, or the Jungle. Educational level was categorized as None (no formal education), primary (grades 1-6), secondary (grades 7-11), or higher (technical or university education). The wealth index, a composite measure of household socioeconomic status calculated by ENDES using principal component analysis of household assets, housing characteristics, and services, was collapsed into three categories: very poor/poor, middle, or rich/very rich (15). Area of residence was classified as urban or rural according to ENDES definitions. Health insurance was defined as the presence or absence of any health insurance coverage. Race or ethnicity was self-identified and categorized as Mestizo, Quechua, Aymara/Native/Indigenous Amazonian/Other, Black/Afro-Peruvian, or White/Caucasian. Obesity was defined as body mass index (BMI) of 30 kg/m² or greater, calculated from directly measured height and weight.

### 2.4 Procedures

ENDES uses various methods to collect information. Initially, paper questionnaires and personal digital devices were used. Since 2016, tablets have been incorporated to streamline the process. Typically, interviews and physical measurements are in person, with trained staff visiting homes. However, due to the COVID-19 pandemic in 2020 and 2021, many interviews were conducted by telephone, and blood pressure measurements were made after the mandatory social isolation ordered by the Peruvian government. For the year 2022, the usual face-to-face process resumed (15).

### 2.6 Statistical analysis

All analyses accounted for ENDES’s complex survey design, including stratification, clustering, and sampling weights, to obtain nationally representative estimates. Statistical analyses were performed using R version 4.3.0 (R Foundation for Statistical Computing, Vienna, Austria) (16).

We first conducted descriptive analyses to characterize the study population, presenting frequencies and weighted proportions for categorical variables. Bivariate associations between independent variables and each outcome were evaluated using the Rao-Scott chi-square test, which adjusts for complex survey design. Multivariable Poisson regression models with robust variance were then fitted to estimate adjusted prevalence ratios (aPR) and 95% confidence intervals (95% CI) for factors associated with each outcome. Separate models were constructed for patients with diabetes and patients with hypertension.

All models were simultaneously adjusted for sex, age group, region of residence, area of residence, educational level, wealth index, health insurance, race or ethnicity, and obesity. Finally, we examined temporal trends by calculating the weighted proportion of patients receiving each check-up annually from 2014 to 2022. These proportions were plotted to visualize changes over time. No formal statistical tests for trend were conducted; therefore, interpretations regarding changes over time are descriptive.

### 2.6 Ethical aspects

This study used publicly accessible, de-identified data from the ENDES survey without direct contact with participants. Therefore, ethics committee review was not required. The manuscript was prepared following the ethical principles of the Declaration of Helsinki.

## 3 Results

The patients evaluated for diabetes and HTN numbered 8,608 and 21,850, respectively. A predominance of female sex was found (HTN: 60.90%, diabetes: 58.18%), older adults (HTN: 71.92%, diabetes: 63.95%), residing in urban areas (HTN: 82.08%, diabetes: 90.44%), and being of high/very high socioeconomic status (HTN: 51.26%, diabetes: 58.46%). Regarding the prevalence of check-ups in the last year, it was high for ophthalmic examinations (HTN 65.46%, diabetes: 70.54%), blood pressure measurements (HTN: 81.82%, diabetes: 79.92%), and glucose measurements (HTN: 56.72%, diabetes: 83.76%) (**Table 1**).

**Table 1:** Sociodemographic and clinical characteristics of Peruvian adults aged ≥50 years with diabetes or hypertension, ENDES 2014-2022.

Characteristic	HTN	Diabetes
	n = 21,850	n = 8,608
Sex		
Male	8,544 (39.10%)	3,601 (41.83%)
Female	13,306 (60.90%)	5,007 (58.17%)
Age group		
50 to 59 years	6,135 (28.08%)	3,104 (36.05%)
60 years to more	15,715 (71.92%)	5,505 (63.95%)
Educational level		
None	107 (0.55%)	18 (0.23%)
Primary	8,159 (41.75%)	2,665 (33.23%)
Secondary	6,205 (31.75%)	3,069 (38.28%)
Higher	5,072 (25.95%)	2,266 (28.26%)
Region of residence		
Metropolitan Lima	8,593 (39.33%)	4,067 (47.25%)
Rest of coast	6,100 (27.92%)	2,437 (28.31%)
Mountain Range	4,699 (21.51%)	1,262 (14.66%)
Jungle	2,458 (11.25%)	842 (9.78%)
Area of residence		
Rural	3,915 (17.92%)	823 (9.56%)
Urban	17,935 (82.08%)	7,785 (90.44%)
Wealth index		
The poorest/Poor	6,512 (29.80%)	1,809 (21.01%)
Medium	4,138 (18.94%)	1,776 (20.63%)
Rich/Richest	11,200 (51.26%)	5,024 (58.36%)
Race		
Mestizo		3,345 (38.86%)
Quechua		1,337 (15.53%)
Aymara/Native/Indigenous Amazonian/Other		2,833 (32.91%)
Negroid/Black		579 (6.73%)
White/Caucasian		514 (5.97%)
Health insurance		
No	3,165 (14.49%)	1,342 (15.59%)
Yes	18,679 (85.51%)	7,267 (84.41%)
Ophthalmic check-up		
< 1 year	14,304 (65.46%)	6,072 (70.54%)
≥ 1 year	7,546 (34.54%)	2,536 (29.46%)
Medical blood pressure check-up		
< 1 year	17,877 (81.82%)	6,880 (79.92%)
≥ 1 year	3,973 (18.18%)	1,728 (20.08%)
Medical check-up		
< 1 year	12,393 (56.72%)	7,211 (83.76%)
≥ 1 year	9,458 (43.28%) ​​	1,398 (16.24%) ​
n (%)		

Source: self-made

**Figure 1:**
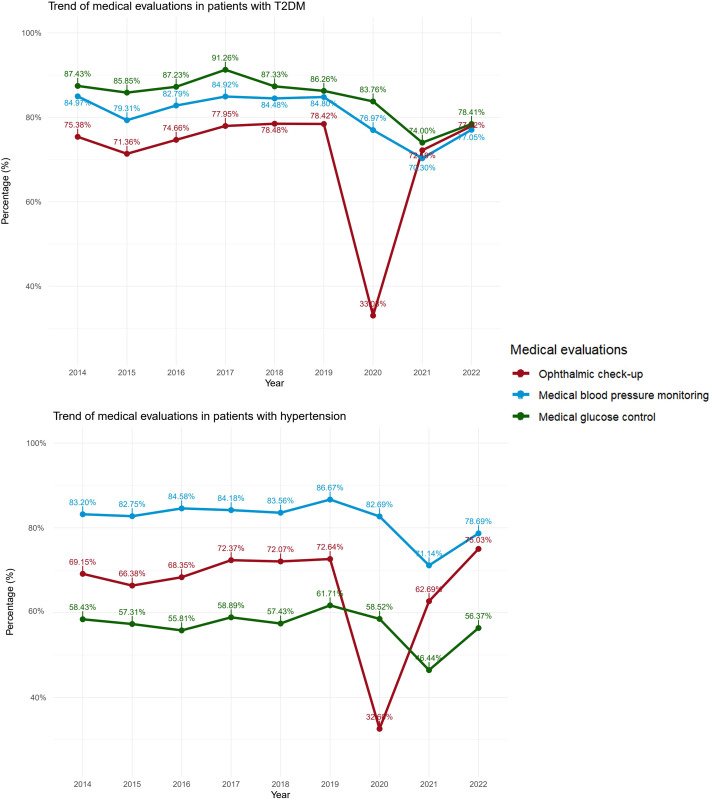
Temporal trends in healthcare check-ups among Peruvian adults aged ≥50 years with diabetes and hypertension, ENDES 2014-2022.

In the trend analysis for patients with diabetes, minimal variation was observed in the evaluations carried out from 2014 to 2019. However, a notable decline was recorded in 2020 and 2021, particularly in ophthalmic check-ups, followed by a recovery in 2022. Similarly, in patients with HTN, a reduction in check-ups during 2020 and 2021 was noted, with a decrease in ophthalmic examinations in 2020, followed by a trend towards recovering these check-ups’ frequency in 2022 (**Figure 1**).

According to the multivariable analysis in patients with diabetes, statistically significant factors for undergoing an ophthalmic check-up in less than one year were age ≥60 years, higher educational level, residing in the jungle region, higher socioeconomic level, and being of Quechua race. For blood pressure check-ups: female sex, age ≥60 years, having health insurance, being of Quechua race, and having obesity. For glucose check-ups, having health insurance was a significant factor (**Table 2**).

**Table 2:** Disparities in healthcare check-ups among Peruvian adults aged ≥50 years with diabetes by sociodemographic characteristics, ENDES 2014-2022.

Characteristic	Ophthalmic check-up		Medical blood pressure check-up		Medical glucose check-up	
	aPR	95% CI	aPR	95% CI	aPR	95% CI
Sex						
Male	—	—	—	—	—	—
Female	1.03	0.97, 1.09	1.05	1.00, 1.11	1.03	0.98, 1.09
Age group						
50 to 59 years old	—	—	—	—	—	—
60 years to more	1.10	1.04, 1.17	1.11	1.05, 1.17	1.01	0.96, 1.07
Educational level						
None	—	—	—	—	—	—
Primary	2.93	1.25, 9.52	0.97	0.60, 1.70	1.04	0.64, 1.86
Secondary	3.10	1.33, 10.1	0.95	0.59, 1.66	1.07	0.66, 1.91
Higher	3.23	1.38, 10.5	1.00	0.62, 1.75	1.07	0.65, 1.90
Region of residence						
Metropolitan Lima	—	—	—	—	—	—
Rest of coast	0.97	0.91, 1.03	1.03	0.97, 1.09	1.03	0.97, 1.10
Mountain Range	0.94	0.86, 1.03	0.97	0.89, 1.06	0.95	0.87, 1.03
Jungle	0.89	0.80, 0.99	1.04	0.94, 1.14	1.01	0.92, 1.11
Area of residence						
Rural	—	—	—	—	—	—
Urban	1.05	0.93, 1.20	1.03	0.92, 1.15	1.02	0.91, 1.14
Wealth index						
The poorest/Poor	—	—	—	—	—	—
Medium	1.25	1.13, 1.38	0.99	0.90, 1.08	1.04	0.95, 1.13
Rich/Richest	1.33	1.21, 1.46	1.05	0.97, 1.14	1.07	0.99, 1.16
Health insurance						
No	—	—	—	—	—	—
Yes	1.04	0.97, 1.13	1.23	1.14, 1.33	1.13	1.05, 1.21
Race						
Mestizo	—	—	—	—	—	—
Quechua	0.91	0.83, 0.99	0.92	0.84, 0.99	0.96	0.89, 1.04
Aymara/Native/Indigenous Amazonian/Other	0.97	0.91, 1.03	1.01	0.95, 1.07	1.01	0.96, 1.07
Negroid/Black	0.91	0.81, 1.02	0.98	0.88, 1.08	0.95	0.86, 1.06
White/Caucasian	0.95	0.85, 1.07	0.95	0.85, 1.06	0.97	0.87, 1.08
Obesity						
No	—	—	—	—	—	—
Yes	1.01	0.95, 1.07	1.06	1.01, 1.12	0.99	0.94, 1.04

*each factor has been independently adjusted for sex, age group, region of residence, area of residence, educational level, wealth index, health insurance, race, and obesity

aRP, Adjusted Prevalence ratio; 95% CI, 95% confidence interval.

Source: self-made.

In the multivariate analysis for patients with HTN, statistically significant factors for undergoing an ophthalmic check-up in less than one year were female sex, age ≥ 60 years, educational level, region of residence, residing in an urban area, socioeconomic status, and having health insurance. For blood pressure check-up: female sex, age ≥ 60 years, residing on the Rest of coast, and having health insurance. For glucose check-up: female sex, age ≥ 60 years, higher educational level, residing on the Rest of coast and in the Mountain Range, urban area, middle and high/very high socioeconomic status, and having health insurance (**Table 3**).

**Table 3:** Disparities in healthcare check-ups among Peruvian adults aged ≥50 years with hypertension by sociodemographic characteristics, ENDES 2014-2022.

Characteristic	Ophthalmic check-up		Medical blood pressure check-up		Medical glucose check-up	
	aPR	95% CI	aPR	95% CI	aPR	95% CI
Sex						
Male	—	—	—	—	—	—
Female	1.04	1.01, 1.07	1.04	1.01, 1.08	1.04	1.01, 1.09
Age group						
50 to 59 years old	—	—	—	—	—	—
60 years to more	1.07	1.03, 1.11	1.10	1.06, 1.15	1.12	1.07, 1.17
Educational level						
None	—	—	—	—	—	—
Primary	1.61	1.18, 2.27	1.12	0.90, 1.42	1.18	0.88, 1.63
Secondary	1.72	1.26, 2.43	1.14	0.92, 1.45	1.33	0.99, 1.83
Higher	1.86	1.37, 2.63	1.19	0.95, 1.51	1.42	1.06, 1.96
Region of residence						
Metropolitan Lima	—	—	—	—	—	—
Rest of coast	0.96	0.92, 1.00	1.06	1.02, 1.10	1.04	1.00, 1.09
Mountain Range	0.90	0.85, 0.95	0.96	0.92, 1.02	0.88	0.83, 0.94
Jungle	0.92	0.86, 0.98	1.04	0.98, 1.10	1.04	0.97, 1.12
Area of residence						
Rural	—	—	—	—	—	—
Urban	1.17	1.08, 1.26	1.03	0.97, 1.09	1.24	1.14, 1.34
Wealth index						
The poorest/Poor	—	—	—	—	—	—
Medium	1.26	1.18, 1.35	1.03	0.97, 1.09	1.15	1.07, 1.24
Rich/Richest	1.39	1.31, 1.48	1.04	0.98, 1.10	1.28	1.20, 1.37
Health insurance						
No	—	—	—	—	—	—
Yes	1.07	1.02, 1.13	1.17	1.12, 1.23	1.31	1.23, 1.39
Race						
Mestizo	—	—	—	—	—	—
Quechua	0.99	0.94, 1.05	0.98	0.93, 1.03	1.00	0.93, 1.06
Aymara/Native/Indigenous Amazonian/Other	1.02	0.98, 1.06	1.04	1.00, 1.08	1.06	1.00, 1.11
Negroid/Black	0.93	0.86, 1.01	1.02	0.95, 1.09	0.95	0.87, 1.03
White/Caucasian	0.96	0.89, 1.03	1.04	0.97, 1.12	1.07	0.98, 1.16
Obesity						
Normal	—	—	—	—	—	—
Obesity	1.03	0.99, 1.06	1.01	0.97, 1.04	1.07	1.03, 1.12

*each factor has been independently adjusted for sex, age group, region of residence, area of residence, educational level, wealth index, health insurance, race, and obesity

aRP, Adjusted Prevalence ratio; 95% CI, 95% confidence interval.

Source: self-made.

## 4 Discussion

### 4.1 Trends in check-ups for patients with HTN and diabetes

Our findings reveal significant disparities in the performance of essential health check-ups, such as ophthalmic examinations, blood pressure measurements, and glucose measurements, among patients diagnosed with HTN or diabetes. Although the prevalence of these check-ups remains high, ranging between 70% and 80%, we have not observed a significant increase in their performance since 2014. This suggests that public health policies focused on promoting awareness of the importance of these check-ups have not effectively expanded their reach. The situation worsened during the pandemic, with a significant decrease in the performance of check-ups, especially ophthalmic examinations. This worrying trend persisted into 2021 despite the relaxation of quarantine measures in Peru, indicating additional barriers for patients to access necessary check-ups.

This phenomenon may reflect not only the limitations imposed by the COVID-19 pandemic regarding access and availability of health services but also pre-existing obstacles related to health infrastructure, the stigma associated with these diseases, and a lack of information or understanding about the importance of regular follow-up (17). Future health policies must address these challenges comprehensively, ensuring that interventions are culturally sensitive, accessible, and tailored to the needs of the most vulnerable populations.

Other studies have found similar situations. Despite clear recommendations for regular monitoring, recent studies have revealed that many patients exceed the year without undergoing the necessary blood pressure, fasting glucose, and Ophthalmic check-ups (7–10).

To improve the situation, it is essential to re-evaluate health communication and education strategies and explore new ways to facilitate access to medical checks. This could include using digital technologies for telemedicine, mobile health programs offering reminders and education on chronic disease management and implementing policies that reduce the financial cost of healthcare for patients.

Adapting health systems to overcome identified barriers and implementing innovative approaches to preventive care is fundamental to ensuring that all patients with diabetes and HTN can regularly and timely perform their necessary medical checks, thereby reducing the risk of complications and improving their quality of life.

### 4.2 Check-ups according to comorbidity

Among patients with diabetes, glucose check-ups were most prevalent, followed by blood pressure check-ups, and finally ophthalmic check-ups, all generally exceeding 70%. However, among patients with HTN, the most frequent check-up was blood pressure measurement, as expected, with 70% for ophthalmic examinations and almost 50% for glucose measurements. This pattern suggests higher regularity in check-ups among patients with diabetes compared to those diagnosed with HTN, even though hypertensive patients are also at risk of developing complications related to hyperglycemia.

A finding that caught our attention is that ophthalmic examinations had the lowest prevalence among the three types of check-ups in patients with diabetes (70.54%), despite the high risk of diabetic retinopathy and vision loss in this population. This discrepancy is especially alarming, as diabetic retinopathy is a leading cause of preventable blindness in working-age adults (18), and early detection through regular ophthalmic examinations can prevent up to 90% of vision loss (19). The lower frequency of ophthalmic examinations compared to blood pressure and glucose measurements may reflect various barriers, such as limited access to ophthalmology services, lack of patient awareness about retinopathy risks, or inadequate referral systems from primary care to specialized ophthalmologic care (20). Faced with this problem, health systems should prioritize strategies to increase ophthalmologic screening rates in diabetic patients, such as integrated care models that coordinate primary care with ophthalmology services, telemedicine screening programs, or point-of-care retinal imaging (21).

The measurement of glucose in patients with HTN, regardless of diabetes diagnosis, is consistent with international guidelines that recommend cardiovascular risk assessment in all hypertensive patients. Current evidence demonstrates that HTN and diabetes share common pathophysiological mechanisms, including insulin resistance and metabolic syndrome. Regular glucose monitoring in hypertensive patients enables early detection of prediabetes or undiagnosed diabetes, which affects approximately 20-30% of hypertensive individuals. This integrated approach to cardiovascular risk management is particularly important in our study population of individuals aged 50 years and older, where the prevalence of undiagnosed dysglycemia is substantial (5, 6).

HTN is a prevalent chronic condition that requires comprehensive health management to address its interconnected complications with diabetes and cardiovascular risk (22). According to medical research, a comprehensive strategy for managing HTN is advocated, as it underscores the interdependence linking elevated blood pressure, diabetes, and jeopardized heart health. For example, Zhang et al. (9) highlight the importance of jointly addressing cardiovascular risk factors to improve health outcomes in populations with chronic diseases. By comprehensively addressing chronic diseases, this coordinated method promises to diminish significantly their worldwide influence while upgrading the well-being experienced by those enduring such conditions. While focusing on blood pressure check-up in hypertensive patients is essential, diligently monitoring and adeptly managing glucose levels is equally crucial.

Evidence indicates that elevated blood sugar and diabetes frequently accompany high blood pressure in patients, notably increasing the chances of developing grave cardiovascular problems and other serious issues stemming from such interrelated health conditions. Therefore, public health strategies and clinical guidelines must reinforce the importance of regular glucose monitoring in patients with HTN to manage HTN better and prevent or manage concomitant hyperglycemia—research like McAlister et al. (23). Demonstrating how judicious glucose regulation can decrease diabetes’ adverse effects highlights that HTN’s management would benefit from paralleling such practices. By accounting for each patient’s unique attributes and circumstances, this holistic methodology aligns with the philosophy of personalized care, where an individual’s specific qualities and requirements are considered when devising customized therapeutic strategies (7, 23).

### 4.3 Associated factors for health check performance

The identification of female sex as a factor associated with higher performance of check-ups in patients with HTN might indicate differences in health perception and behavior between men and women. Studies have revealed a tendency among females to demonstrate greater proactivity concerning their well-being by more regularly pursuing medical attention and adhering to preemptive healthcare guidance than males (24–26). This could reflect a greater awareness of or concern for personal health or differences in socialization that encourage a greater willingness to seek help and adhere to medical advice. Additionally, women often assume caregiver roles in the family, which could translate into greater responsibility and attention to their health to care for others (27, 28).

A heightened cognizance of the dangers of diabetes and HTN issues as one reaches sixty years old may lead to stricter conformity to clinical administration among the older generation (29, 30). With advancing years, often comes a lengthier medical history confronting chronic conditions, which may cultivate a more decisive impetus to participate in preventive and regulatory undertakings. Furthermore, this group may interact more with the health system due to other age-related conditions, thus facilitating their participation in regular check-ups (31, 32).

The link between higher educational levels and increased performance of check-ups suggests that education can play a crucial role in promoting healthy behaviors. Those with greater educational attainment may more readily grasp the significance of proactively directing their wellness, including the necessity for consistent checkups regarding their state. Through education, individuals can significantly improve their capacity to independently obtain applicable healthcare knowledge, accurately comprehend such information, and suitably respond. This is consistent with studies that have found education to be associated with better diabetes self-management and greater adherence to treatment (33–35).

Living in urban areas is commonly associated with better access to health services, including facilities for regular checks of chronic conditions. Urban areas typically offer a higher density of healthcare providers and facilities, from clinics and hospitals to specialized centers for chronic disease management (9). Additionally, these areas may provide greater availability of health information and education programs, making urban residents better informed about the need and benefits of regular check-ups. However, it’s essential to acknowledge that while urbanization can facilitate access to health services, it can also present unique barriers, such as congestion and high living costs, that may influence individuals’ ability to seek care.

A higher socioeconomic standing has been linked to augmented check-up execution, which could stem from improved admission to healthcare assets and decreased financial obstacles to obtaining treatment. Those with more significant financial means may encounter fewer challenges in paying the expenses linked to medical care, such as doctor appointments, lab work, and prescribed drugs, than those with fewer economic resources (7, 36). Moreover, they might have better access to quality health information and preventive healthcare services.

An interesting finding is the relationship between obesity and the performance of these checks. Although patients with obesity show a slight increase in the frequency of checks compared to those without, this increase is not substantially significant. This result is striking since, intuitively, one might expect that the condition of obesity, being a known risk factor for complications in diseases such as diabetes and HTN, would motivate greater vigilance and control. Existing literature has found obesity to be a significant risk factor for numerous health conditions, including cardiovascular diseases, type 2 diabetes, and certain types of cancer (37, 38). Given this association, it would be logical to assume that obese individuals would be more inclined to participate in regular checks to monitor their health. However, our study suggests that obesity does not guarantee greater adherence to recommended check-ups. This could indicate that there are additional barriers for these patients, such as stigma associated with obesity, lack of resources, or even a possible underestimation of personal risk that prevents more active participation in managing their health (39).

It’s crucial to recognize and address the barriers that obese patients face in accessing preventive care and regular check-ups. Literature suggests that weight-related stigma in the healthcare context can discourage obese individuals from seeking preventive care and participating in regular check-ups (39–41). Additionally, public health interventions promoting the importance of check-ups should be designed inclusively, ensuring that specific barriers preventing obese patients from seeking and receiving the care they need are addressed and minimized.

The finding that Quechua ethnicity patients have a lower likelihood of performing health check-ups, primarily ophthalmic examinations and blood pressure measurements, in the context of diabetes but not in patients with HTN raises essential questions about inequalities in access and utilization of health services among different ethnic groups. This pattern suggests that barriers to accessing check-ups are not uniform across all chronic conditions or population groups (7, 36). The absence of significant differences in check-ups among patients with HTN indicates that the factors limiting the performance of check-ups in the Quechua population might be more related to specific characteristics of diabetes management or particular cultural perceptions and practices related to this disease. The lower likelihood of performing check-ups in the Quechua population may be due to multiple factors, including linguistic barriers, lack of resources, geographical distances to health centers, and possibly, the perception of discrimination or negative previous experiences in the health system (42). Additionally, cultural aspects may influence the perception of the need and utility of regular check-ups (43).

Health policies must recognize and address these ethnic disparities in access to healthcare. However, the suggestion that health policies do not prioritize certain ethnicities should be approached with caution and, instead, focus on how to adapt and improve the accessibility and cultural relevance of health services to ensure that all population groups, including indigenous communities like the Quechua, can access adequate check-ups and treatments.

### 4.4 Health system context in Peru

The disparities documented in our study must be understood within the context of Peru’s fragmented health system. Peru operates two main public insurance subsystems: the Seguro Integral de Salud (SIS), covering approximately 62% of the population (primarily low-income individuals and vulnerable groups), and the Seguro Social de Salud (EsSalud), covering 30% (formal sector workers and their dependents) (44, 45). While insurance coverage reached 97% by 2023 following the 2009 Universal Health Insurance Law, substantial barriers to accessing quality care persist (46). These subsystems operate separate provider networks with limited integration, creating inefficiencies and access gaps, particularly in rural areas (47).

A critical challenge is the uneven distribution of health infrastructure. The most recent evaluation by the Ministry of Health indicates that over 90% of SIS primary care facilities are precarious, obsolete, or insufficiently equipped (48). In contrast, EsSalud facilities provide better access to specialized services and equipment. This infrastructure disparity partly explains our finding that individuals with health insurance and higher socioeconomic status have better check-up completion rates: they are more likely to be affiliated with EsSalud or access better-equipped private facilities through out-of-pocket payments. The concentration of healthcare resources in urban and coastal areas (46) directly corresponds to the geographic disparities we observed, with rural and highland residents having significantly lower rates of ophthalmic and glucose check-ups.

The system’s fragmentation also creates barriers for continuity of care and referrals between primary care and specialized services, which may particularly affect access to ophthalmological examinations requiring specialized equipment and personnel. Despite Peru’s progress toward universal coverage, the country’s health expenditure remains below regional standards (6.2% of GDP in 2022), resulting in resource constraints that disproportionately affect SIS facilities and the populations they serve (46). These structural characteristics of Peru’s health system provide essential context for understanding why socioeconomic, geographic, and insurance-related disparities in healthcare utilization persist despite near-universal insurance coverage.

### 4.5 Contribution to the field

The study’s discoveries hold immense relevance for societal well-being, specifically regarding bettering the handling and regulation of prolonged afflictions like diabetes and high blood pressure through optimized protocols informed by these results. Firstly, identifying demographic and socioeconomic factors associated with higher performance of check-ups underscores the need to develop public health strategies that are inclusive and accessible to all population segments. This passage emphasizes the importance of removing obstacles hindering particular demographics like males and individuals residing in rural locales from routinely undergoing health evaluations.

The evidence of diminished check-up performance during the pandemic, with its implications for vulnerabilities within health systems when faced with global health crises, highlights the necessity of sustaining fundamental health services throughout such difficult times. This suggests that health systems need to be resilient and flexible, capable of adapting to changing circumstances to ensure the continuity of care for patients with chronic conditions.

The association with social factors also suggests that public health interventions must go beyond physical access to health services. Implementing health education programs that improve disease understanding and promote proactive health behaviors across the population, regardless of patient characteristics, is crucial.

Ultimately, the study underscores the need for governments to adopt policies focused on establishing universal healthcare systems and equitable availability of high-standard medical care for all people within a society, irrespective of financial means. Providing equitable access to routine preventive healthcare and adequate self-management support for chronic illnesses across all demographics, including differences in gender, age, location, or financial circumstance, is imperative to enhance a population’s overall health status and narrow the gaps in health that unjustly persist between subgroups.

### 4.6 Study limitations

Several methodological limitations should be considered when interpreting our findings. While the study’s cross-sectional design does not allow for causal inference between identified factors and healthcare utilization, this limitation does not affect our primary objective, which is to describe disparities in healthcare check-ups across different population subgroups rather than to establish causal relationships. The reliance on self-reported data may be subject to recall bias or social desirability bias, potentially affecting the accuracy of information related to performing medical check-ups and the presence of chronic conditions. Additionally, the survey does not differentiate between comprehensive ophthalmological examinations and basic optometric evaluations; however, our study focuses on disparities in access to eye care services rather than the specific type of examination received, and the primary concern remains that many patients are not accessing any form of eye evaluation. Our analysis was restricted to individuals aged 50 years and older because ENDES collects data on ophthalmic examinations systematically only from this age group, excluding younger individuals with diabetes or hypertension, though this age restriction reflects both the survey’s design and clinical prioritization of retinopathy screening in older populations with longer disease duration and higher complication risk. The analysis could not capture all factors that may influence healthcare utilization, such as personal health beliefs, stigma associated with chronic conditions, or quality of patient-provider relationships. Finally, no formal statistical tests for temporal trends were conducted; therefore, interpretations of changes over time are descriptive.

An important consideration is the composition of our study sample. While ENDES is representative of the general Peruvian population, our analysis was restricted to individuals with self-reported physician diagnoses, introducing detection bias: individuals from higher socioeconomic strata have greater healthcare access and are more likely to be diagnosed and aware of their condition, resulting in their overrepresentation (51-58% in the highest wealth quintiles). Conversely, individuals from lower socioeconomic strata likely have substantial undiagnosed diabetes and hypertension due to limited healthcare access, leading to their underrepresentation in our sample. This detection bias means our findings reflect disparities among those aware of their diagnosis while missing potentially larger inequities among undiagnosed individuals. Therefore, our results likely underestimate the true magnitude of healthcare access disparities in the Peruvian population with these conditions. Additionally, the ability to generalize results may be limited by cultural, economic, and healthcare system differences, meaning findings may not directly apply to contexts outside Peru.

## 5 Conclusions

While this research uncovered meaningful inconsistencies in how well Peruvians with diabetes or hypertension completed necessary health check-ups, it also demonstrated that variables including age, education, socioeconomic class, and access to medical insurance greatly impacted how often these checks occurred. Although chronic conditions remain widespread, the findings indicate that current efforts to boost health awareness and promotion have not proven adequately successful in encouraging the more consistent monitoring of such vital screenings.

Given the uncovered evidence, strengthening current public health policies and targeting awareness campaigns towards highlighting the necessity of consistent surveillance of such illnesses, especially for demographics found to be more susceptible, would be the prudent path forward. Developing and promoting educational programs focused on the importance of regular checks and how they can prevent serious complications is essential. To promote equitable healthcare for all communities, experts recommend both enhancing the accessibility of insurance coverage and broadening primary care services so that regular, high-quality clinical evaluations are available to every individual irrespective of their residence, economic circumstances, or education. Lastly, implementing more resilient health systems that can maintain continuity of preventive and follow-up care during health crises is crucial, ensuring that patients with chronic conditions receive the necessary care without significant interruptions.

## Data Availability

The data presented in this study comes from the Demographic and Family Health Survey (ENDES), which are deposited on the platform of the National Institute of Statistics and Informatics (INEI) and can be accessed through the following link: https://proyectos.inei.gob.pe/microdatos/ (accessed on January 12, 2025).
